# A Global Approach to Modeling Injection Molding

**DOI:** 10.3390/polym16010147

**Published:** 2024-01-03

**Authors:** Przemysław Narowski, Krzysztof Wilczyński

**Affiliations:** Polymer Processing Department, Faculty of Mechanical and Industrial Engineering, Warsaw University of Technology, Narbutta 85, 02-524 Warsaw, Poland; przemyslaw.narowski@pw.edu.pl

**Keywords:** injection forming, polymeric materials, modeling, plasticizing

## Abstract

A problem of modeling plastic injection forming (molding) is presented, including both the plasticizing system of the injection-forming machine and the mold. When modeling the plastic flow in the mold, the input quantities are essentially unknown, e.g., the plastic melt temperature. Thus, a comprehensive (global) model of the injection-forming process is needed for the flow in the plasticizing system and in the mold. The process output quantities from the plasticizing system will be the input quantities for the mold. When modeling the plastic flow in the injection-forming machine, a comprehensive approach should be applied to consider the solid material conveying, material plasticizing, and the material melt flow. The model of material plasticizing is a basis for building such global models. In this research, the effect of the flow (including plasticizing) in the injection-forming machine on the flow in the mold is studied by simulation (using Moldex3D 2023R3OR 64-bit software) and experimentation. These studies are carried out for the injection forming of selected material using a specialized spiral mold. Simulations performed with the use of Moldex3D software for the plasticizing system significantly improved the accuracy of the simulation of the flow in the mold. However, the best results were obtained using experimental data (plastic melt temperature) as input quantities for mold filling simulations. The novel concepts of injection-forming process modeling based on our previous experimentations are also discussed.

## 1. Introduction

**Figure 1:**
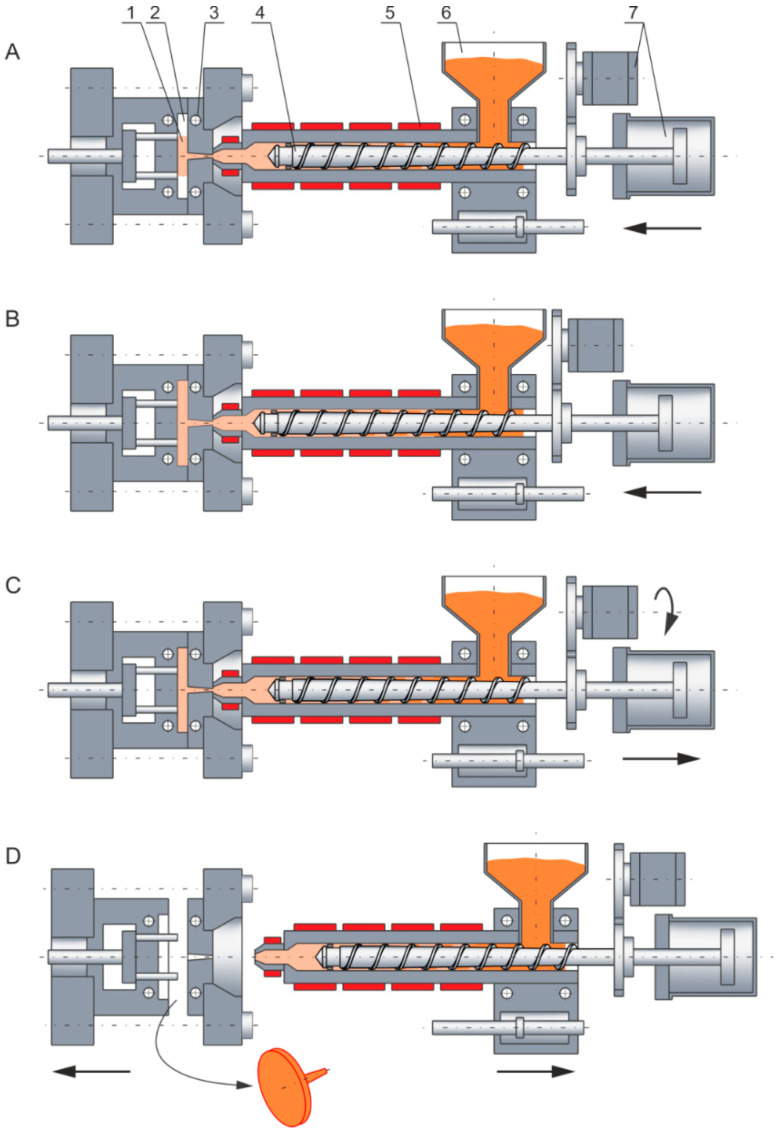
Injection forming: (**A**)—injection (filling), (**B**)—holding (packing), (**C**)—plasticizing (melting), (**D**)—mold opening (part ejection); 1—part (molding), 2—mold cavity, 3—cooling channels, 4—screw, 5—heaters, 6—hopper, 7—drive system (with permission from Rheology in Polymer Processing. Modeling and Simulation by K. Wilczyński; Carl Hanser Verlag: Munich 2021 [1]).

Extrusion and injection forming (molding) are important techniques in the plastic processing industry. Extrusion and injection forming are somewhat similar. In both cases, plasticizing of the material is carried out in the plasticizing system (plasticating unit) of an extruder or injection-forming machine, and the transport of the material to the forming tool is carried out using a screw. Plasticizing (melting) results from the material heating up by the heaters and energy dissipation. However, there are also basic differences between these techniques. Extrusion is a continuous process and involves pushing (extruding) the material through a forming tool, i.e., a die, while injection forming is a cyclic process and involves injecting the material into a closed forming tool, i.e., a mold. In the extrusion process, the screw only rotates, while in the injection-forming process, it performs rotational and reciprocating motions (in the injection phase, the screw acts as a piston). The injection-forming process is presented in Figure 1. It is seen that the screw rotates, the molten material is conveyed to the screw front, and it is injected into the mold through an axial movement of the screw.

Nowadays, plastic processing design is supported by computer modeling. Simulations enable us to predict the process flow based on the processing data, i.e., material properties, injection-forming operating data, and the geometry of the machine and the tool. When modeling injection forming and extrusion, the comprehensive (global) approach should be applied to consider the solid material transport and material plasticizing (in the plasticizing system), as well as the material melt flow (in the plasticizing system and the forming tool, i.e., in the die or the mold). The model of material plasticizing is a basis for building such global models. When modeling the plastic flow in the mold, the input quantities are essentially unknown, e.g., the plastic melt temperature. Thus, a comprehensive model of injection forming is needed for the flow both in the plasticizing system and the mold. The output quantities from the plasticizing system will be the input quantities for the mold.

**Figure 2:**
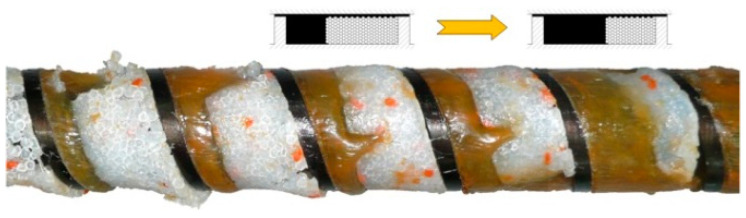
The plasticizing phenomenon at single-screw extrusion with flood feeding (PP) [12].

Modeling research for screw processing of plastics was carried out first for extrusion. The issue of modeling this was presented, in detail, by Wilczyński et al. [2]. The first flow analyses for single-screw extruders were performed by Carley et al. [3,4], who modeled the Newtonian drag/pressure flows. Solid material transport was considered first by Darnell et al. [5], who modeled the material conveying and pressure build up. The first plasticizing research for single-screw extruders was made by Maddock et al. [6,7], who applied the “screw pulling out technique” to study the plasticizing phenomenon. They observed that the molten material accumulated at the active screw flight, and the solid material gradually diminished by the effects of heat conduction from the cylinder and viscous dissipation in the molten plastic (Figure 2). Tadmor et al. [8,9,10] made similar investigations and built the first model of material plasticizing in extrusion, which enabled us to build the first computer model of extrusion of plastics, i.e., EXTRUD [11].

The classical single-screw extrusion with flood feeding was extensively investigated, but little information was available on the extrusion with metered feeding, and the first study was made by Lopez-Latorre et al. [13], and later by Isherwood et al. [14] and Thompson et al. [15]. Recently, Wilczyński et al. [12,16,17] carried out extensive research on this technique and presented the mechanism and model of material plasticizing. Then, they developed the first computer programs to simulate this process, i.e., SSEM-Starve [18,19,20].

Investigations of plasticizing in the twin-screw machines were much more limited. Modeling a twin-screw extrusion process was discussed, in detail, by Lewandowski et al. in a review paper [21], as well as Vergnes [22]. It is important to note that this process is carried out with metered feeding, mainly starvation.

The concepts of research and modeling the material transport and plasticizing in the extrusion process were adopted to model them in the injection-forming process. But, while many studies have referred to modeling the extrusion process, there were fewer on the injection forming. These were mainly limited to modeling the flow in the molds and did not consider the material transport and plasticizing in the plasticizing system; for example, Manzione et al. [23], Isayev et al. [24], Osswald et al. [25], Kennedy et al. [26], and Wang et al. [27]. Some books referred to the tool design; for example, Menges et al. [28], Rees [29], Unger [30], Mennig et al. [31], Turng et al. [32], Beaumont [33], Catoen et al. [34], and Kazmer [35], or the injection-forming machines, such as Johannaber [36]. A valuable review paper on the issues of the simulation of the injection-forming process was written by Fernandes et al. [37]. Nowadays, the most valuable programs for simulating injection forming are the MOLDFLOW program [38], Moldex3D program [39], and CADMOULD program [40], which were recently applied in [41,42]. Simulations were also carried out with the use of CFD software COMSOL Multiphysics [43,44].

Recently, Wilczyński et al. discussed, in detail, the modeling of the injection-forming process in a review paper [45] and performed experiments to discuss the existing models and their physical basics. In their research, the “screw pulling-out technique” was used to analyze the material conveying and plasticizing. Based on these analyses, novel approaches to modeling were presented and discussed.

The first experiments on plasticizing in the injection-forming machines were made by Donovan et al. [46], who used the “screw pulling out technique”. They noticed that the screw recharge was the transient plasticizing process, which gradually approached the equilibrium extrusion as the screw rotated. If screw rotation accounted for a large portion of the whole cycle time, plasticizing was similar to the extrusion, but if the screw rotation accounted for a small portion of the whole cycle time, plasticizing was significantly different.

Valuable experimental research on the material plasticizing in the injection-forming machines was performed by Gao et al. [47,48], who built a visual system to study this. They concluded that plasticizing in the injection-forming process cannot be treated like in the extrusion process. It was noticed in these studies that there was more material plasticized at a lower screw rotation than at a higher rotation. A lower screw rotation resulted in a longer plasticizing time, while a higher screw rotation resulted in a shorter plasticizing time. It was also observed that for a lower plasticizing stroke, the plasticizing was faster, while for a higher stroke, the plasticizing was slower. Moreover, it was noticed that an increase in backpressure was advantageous to plasticizing. An increase in the rest time resulted in more heat being conducted, and more material was plasticized. Other visual investigations were carried out by Pham et al. [49], who confirmed the classical theories and observations.

Donovan [50,51,52] developed the first model for material plasticizing in the injection-forming machine. This required the use of an empirical parameter, specific to a particular material, over the investigated range of process data. Lipshitz et al. [53] developed a model for plasticizing based on the analyses of physical phenomena observed in the injection-forming process. Later, several papers were published on modeling the material plasticizing in the injection-forming machines, e.g., Rauwendaal [54,55], Dormeier and Panreck [56], Rao [57], and Potente [58,59].

Bereaux et al. [60] considered the screw as a pump that conveys a solid material and a molten material. This two-phase conveying has been considered as one-phase conveying by assuming that the pressure gradient was due to melt conveying, but the flow rate at any cross-section was the sum of the solid material flow rate and the melt flow rate.

Yung et al. [61,62,63] discussed a process that was composed of three steps: plasticizing (screw rotating and moving backward), stopping (no screw motion), and mold filling (screw moving forward without rotation). When looking at Figure 1 we can distinguish A—a screw moving forward without rotation, i.e., filling (injection), B—no screw movement, i.e., stopping (holding), C—a screw rotating and moving backward, i.e., feeding (plasticizing), and D—no screw movement, i.e., stopping (molding ejection).

A comprehensive approach to modeling the plasticizing in injection forming that reflected the dynamics of the process was built by Steller and Iwko [64,65,66]. Another concept was applied by Fernandes et al. [67], who modified the extrusion model built by Gaspar-Cunha [68].

As a result of the literature and the review papers [2,45], there are many computer systems used for simulating the extrusion, such as the EXTRUD system [69], the SPR system (Scientific Process & Research) [70], the NEXTRUCAD system [71], the REX system (Rechnergeschützte Extruderauslegung) [72,73], the PASS system (Polymer Analyses and Simulation Software) [74], the SSEM system (Single-Screw Extrusion Model) [75], and the models proposed by Fukase et al. [76], Zavadsky et al. [77], Vincelette et al. [78], and Amellal et al. [79]. But the only available system for injection forming is the PSI system (Paderborner Spritzgiesssimulation) [80].

Recently, a novel version of the Moldex3D 2023R3OR 64-bit software has been developed which allows us to simulate the flow in the plasticizing system (polymer melting and polymer melt flow) [39]. The computational models used there were not presented; however, they were based on the models developed by Tadmor [8], Chang et al. [81], and Altinkaynak et al. [82].

In summary, a few existing models of the injection-forming process (plasticizing system) are based on the studies of the extrusion process and were tested by measurements of pressure or temperatures. But the solid bed profile was not evaluated.

Donovan [50] investigated the injection-forming process using the “screw pulling-out technique”, but the photographs of the screws were not shown. There is a lack of research on the material conveying and plasticizing in injection-forming machines. The reason is that the “screw pulling out” experiments for injection forming are extremely difficult. It is difficult to quickly remove the screw from the machine to prevent the material from plasticizing.

Therefore, Wilczyński et al. [45] carried out the experiments to discuss the existing models of injection forming and their physical bases. In these experiments, the “screw pulling-out technique” was used to study the material conveying and plasticizing. Based on this research, a novel approach to modeling has been presented. It was observed that starving appeared when the screw moved forward, filling the material into the mold. This was dependent on the screw rotation, plasticizing stroke, and backpressure.

**Figure 3:**
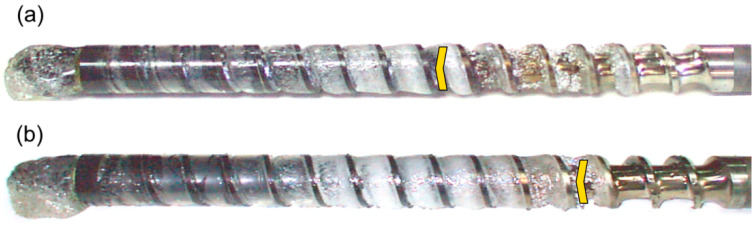
The effect of screw speed on polymer flow in the injection molding process at the plasticization stroke h_plast_ = 1D and the backpressure P_back_ = 0 MPa: (**a**) N = 100 rpm, (**b**) N = 300 rpm [45].

An example of the results of these studies is shown in Figure 3. An influence of the screw rotation (N = 100 rpm and N = 300 rpm) on the material conveying in the injection-forming machine at a constant plasticizing stroke and constant backpressure is presented. It is observed that when the screw rotation increases, plasticizing slows down, which results from the shorter plasticizing time, and is consistent with Gao’s observation [47]. It is also observed that starving decreases since more material is supplied to the front of the screw tip (a yellow mark indicates starving).

**Figure 4:**
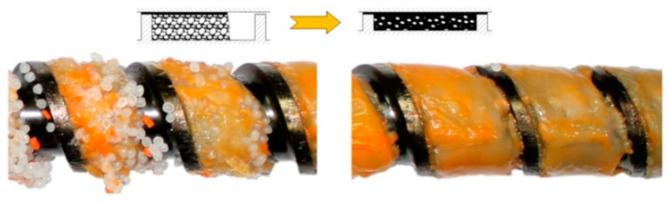
The plasticizing phenomenon at single-screw extrusion with metered feeding (PP) [12].

In general, the existing models of the injection-forming process (plasticizing system) discussed are different from the extrusion models in that they involve the static/dynamic phases of plasticizing. But it is assumed that the screw is fully filled with a material, like in the extrusion with flood feeding (Figure 2), which is inconsistent with our studies [45] where starving was observed, like in the extrusion with metered feeding (Figure 4).

In this paper, we have performed simulations of the plastic flow in the plasticizing system and mold to evaluate the influence of the input plastic temperature on the accuracy of simulations of the flow in the mold. Simulations have been supported and verified by experimentation.

## 2. Research Program

### 2.1. Experiment

**Figure 5:**
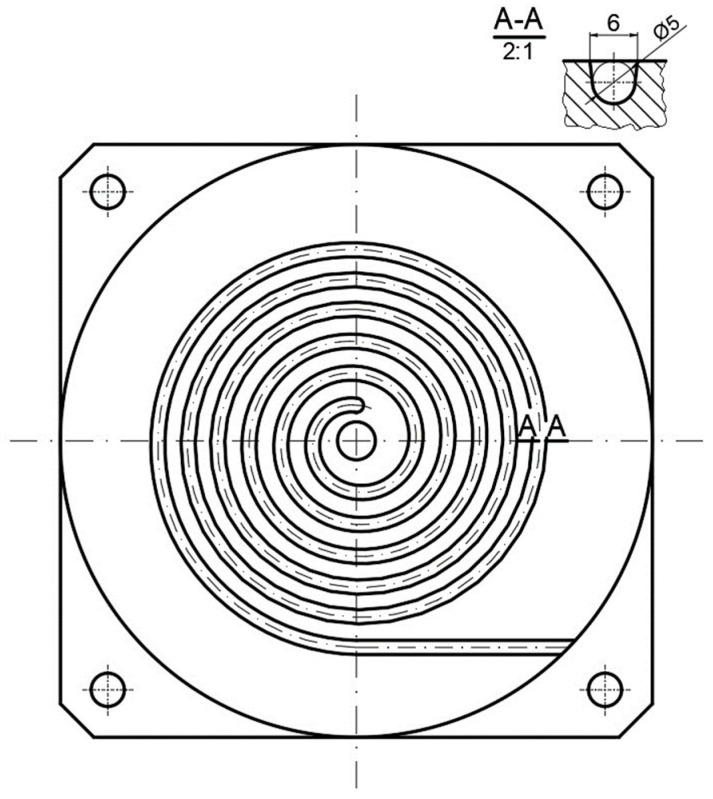
Spiral mold.

Experiments have been carried out to investigate the influence of the process operating conditions, screw rotation, and backpressure on the temperature of the material leaving the injection-forming machine and mold filling. Experiments have been carried out using a specialized spiral mold (Figure 5), and the length of the spiral molding was measured. The polymer melting temperature of the material flowing from the injection molding nozzle was measured using the thermal imaging camera FLUKE Ti480 PRO, manufactured by Fluke Corporation, Everett, WA, USA.

The injection-forming machine FORMOplast 235/80 (produced by PONAR, Żywiec, Poland) was used with a clamping force of F_clamp_ = 80 T. A three-sectional screw with a diameter of D = 35 mm and length L = 735 mm; that is, an L/D ratio equal to L/D = 21 and a screw pitch of t = 35 mm was applied. The screw section lengths were equal to L_F_ = 385 mm (feeding section), L_C_ = 210 mm (compression section), and L_M_ = 140 mm (metering section). The screw channel depths in the sections were equal to H_F_ = 6 mm (feeding section) and H_M_ = 2.4 mm (metering section), and the compression ratio was equal to CR = H_F_/H_M_ = 2.5.

### 2.2. Process Simulations

Simulations have been performed to investigate the influence of the process operating conditions, screw rotation, and backpressure on the temperature of the material leaving the injection-forming machine and the influence of this temperature on the filling of the spiral mold used in the experiment (Figure 5). The length of the spiral has been evaluated.

Simulations have been carried out using Moldex-3D (2023R3OR) software developed by CoreTech System Co., Ltd. (Zhubei, Taiwan). This program is capable of simulating the flow in the mold and the plasticizing system. It allows us to calculate the profiles of bulk temperature, pressure, shear rate, shear stress, solid bed ratio, relative un-melted polymer amount along the screw, etc. However, the modeling basics are not presented in the literature [39].

The whole model for mold filling has been meshed with 1,887,778 3D solid mesh elements (Tetra, Pyramid, Prism, and Hexa). Mold cooling and filling simulation lasted for about 8.5 h using a Lenovo ThinkPad P15 Gen 2i computer equipped with a 2.50 GHz processor and 32 GB of RAM.

Three approaches to modeling the plastic melt flow in the injection mold have been tested, which differ in the way of determining the temperature of the plastic melt flowing into the mold:

A—Common approach, i.e., simulations based on the recommended injection temperature given in the plastic material card;

B—Simulation approach, i.e., simulations based on the material temperature at the exit of the barrel of the injection-forming machine, obtained as a result of Moldex3D computations;

C—Experimental approach, i.e., simulations based on the material temperature at the exit of the barrel of the injection-forming machine, obtained as a result of the experiment.

### 2.3. Material and Process Data

**Figure 6:**
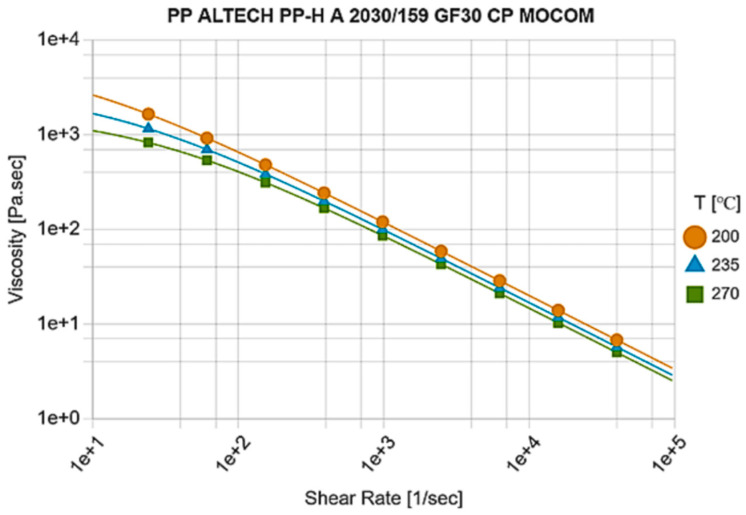
Viscosity curve [39].

The polypropylene PP GF30 (Altech PP-H A 2020/159 GF30 CP, manufactured by MOCOM Compounds GmbH & Co. KG, Hamburg, Germany) was applied. The viscosity curve of the material is shown in Figure 6.

The viscosity was described using the Cross-WLF equation [39], which may have the following form:

(1) $$\mu =\frac{\mu_{0}}{1+{\left(\frac{\mu_{0}\dot{\gamma }}{\tau^{C}}\right)}^{1-n}}$$

(2) $$\mu_{0}=D_{1}exp[-\frac{A_{1}\left(T-T^{*}\right)}{A_{2}+\left(T-T^{C}\right)}]$$

where μ is viscosity, Pa∙s; $\mu_{0}$ is zero viscosity, Pa∙s; $\dot{\gamma }$ is the shear rate, s^−1^; $\tau^{C}$ is critical shear stress, Pa; $\mu_{0}$/$\tau^{C}$ is relaxation time λ, s; *n* is the power law parameter; *T* is the temperature, K; and *D*_1_, *A*_1_, *A*_2_, and *T^C^* are the Cross-WLF model parameters.

Different operating parameters were used to investigate their effect on material conveying and plasticizing in the injection-forming machine and the mold. The screw rotations were equal to N = 55 rpm, 110 rpm, 175 rpm, 240 rpm, and 310 rpm, the plasticizing stroke was constant at h_plast_ = 1.5D, and the backpressures (hydraulic) were equal to P_back_ = 17 MPa, 34 MPa, and 51 MPa. The temperatures were set at T_I_ = 190 °C, T_II_ = 200 °C, and T_III_ = 220 °C in the cylinder sections and T_nozzle_ = 230 °C in the nozzle.

The process and material parameters [39] are collected in Table 1.

## 3. Results

### 3.1. Plastic Flow in the Plasticizing System

**Figure 7:**
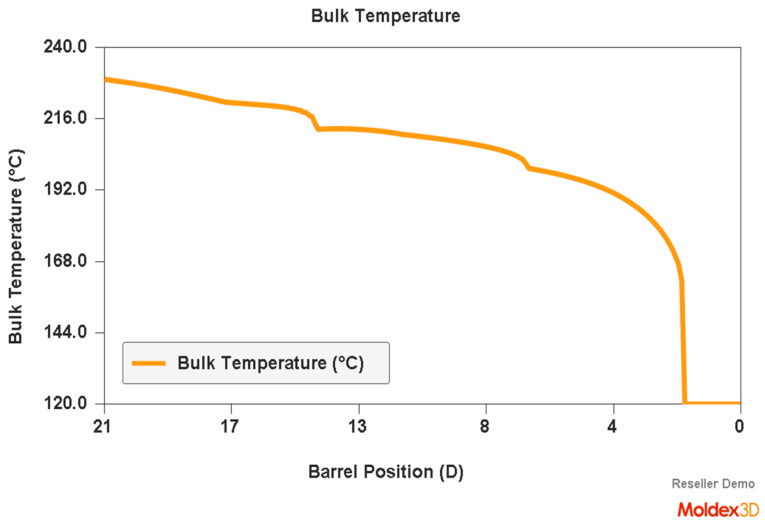
Simulation of plastic flow in the injection-forming machine (bulk temperature) obtained at a screw rotation speed of N = 175 rpm and a backpressure of P_back_ = 34 MPa.

**Figure 8:**
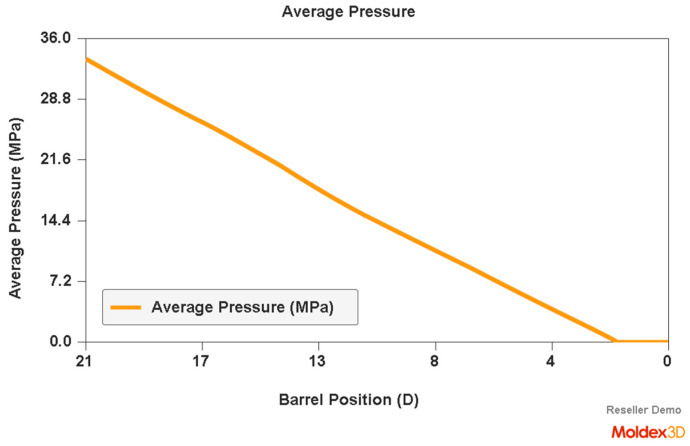
Simulation of plastic flow in the injection-forming machine (pressure) obtained at a screw rotation speed of N = 175 rpm and a backpressure of P_back_ = 34 MPa.

**Figure 9:**
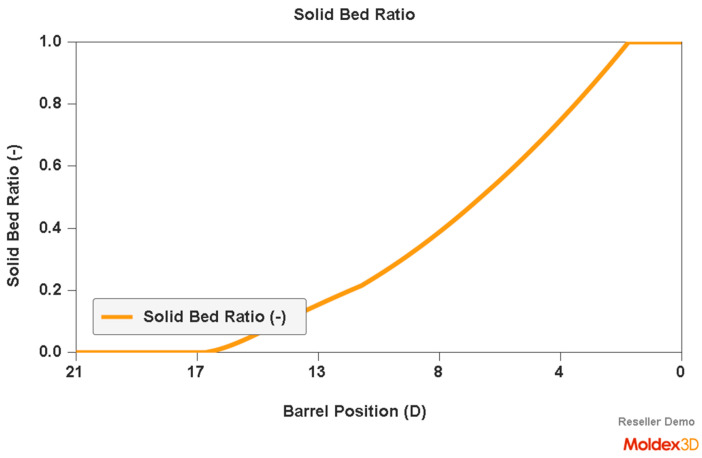
Simulation of plastic flow in the injection-forming machine (solid bed profile) obtained at a screw rotation speed of N = 175 rpm and a backpressure of P_back_ = 34 MPa.

An example of a simulation of the plastic flow in the injection-forming machine at a screw speed N = 175 rpm and a backpressure of P_back_ = 34 MPa is shown in Figure 7, Figure 8 and Figure 9. The profiles of bulk temperature, pressure, and solid bed ratio along the screw are presented. The temperature computed at the end of the barrel has been used for the simulation of the filling of the spiral mold under study.

### 3.2. Plastic Melt Temperature

**Figure 10:**
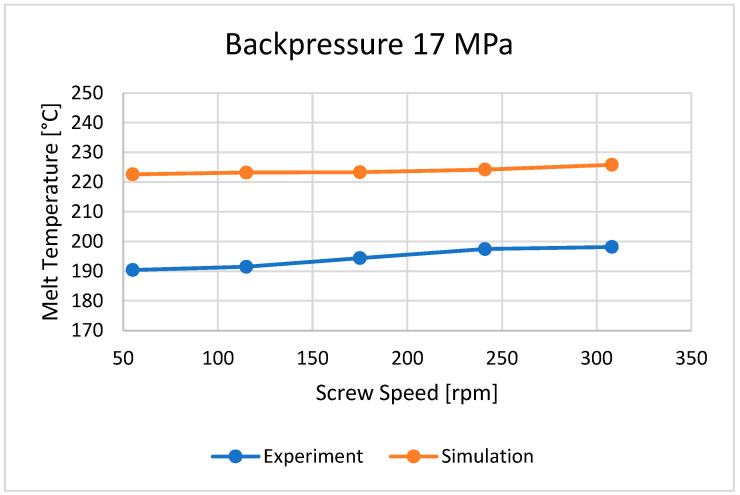
The influence of screw rotation N on the melt temperature at a backpressure of P_back_ = 17 MPa.

**Figure 11:**
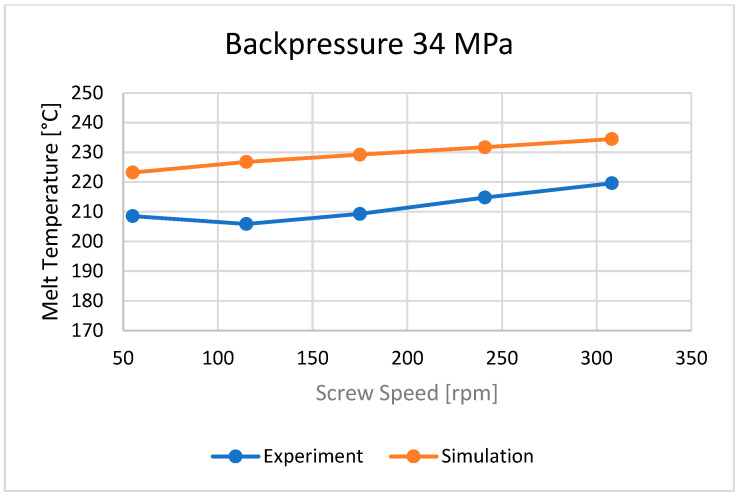
The influence of screw rotation N on the melt temperature at a backpressure of P_back_ = 34 MPa.

**Figure 12:**
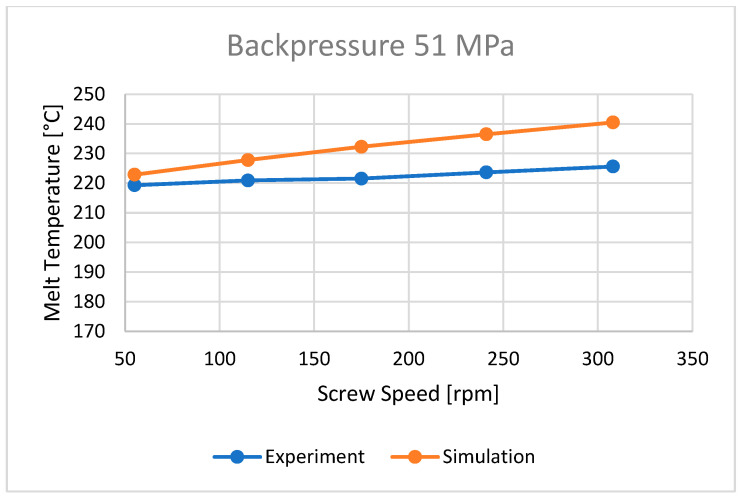
The influence of screw rotation N on the melt temperature at a backpressure of P_back_ = 51 MPa.

The bulk temperature calculated at the end of the barrel was compared with the results of measuring the temperature of the material leaving the injection-forming machine, i.e., flowing from the nozzle (Figure 10, Figure 11 and Figure 12). It is seen that the plastic melt temperatures, both calculated and measured, increase with an increase in the screw rotation (to a relatively small extent) and an increase in backpressure. In each case, the calculated temperature is higher than the measured temperature, which means that the calculations overestimate the temperature of the material.

The plastic flows in the injection molds and plasticizing systems are complex due to the non-Newtonian behavior and high rates of shearing. An energy dissipation (heat generation) in the flow is determined by two factors, shear rate and viscosity, according to:

(3) $$E=\eta \left(\dot{\gamma },\;T\right){\dot{\gamma }}^{2}$$

where *E* is energy dissipation in time in the volume element, J/(m^3^·s) = W/m^3^; η is viscosity, Pa·s; $\dot{\gamma \;}$ is the shear rate, 1/s; and *T* is the temperature, °C.

An increase in the shear rate diminishes viscosity, thus diminishing heat generation, but this also increases heat generation (approximately to the second power). Which of these factors prevails is dependent on the plastic rheological characteristics. When screw speed increases, the shear rate also increases; however, at the same time, the material residence time in the plasticizing system diminishes, and thus the heat delivered to the material by conduction from the heaters diminishes. Moreover, an increase in screw speed slows down the plasticizing process, and the length of plasticizing increases.

According to Gao’s observations [47,48], the backpressure influences plasticizing, pressure, and temperature distributions. It was observed that an increase in backpressure was advantageous to plasticizing. When backpressure increases, the screw cannot easily retreat, and the contact time between the material and the screw increases, generating more shear heat (energy dissipation), which causes the material temperature to rise.

### 3.3. Flow Length

**Figure 13:**
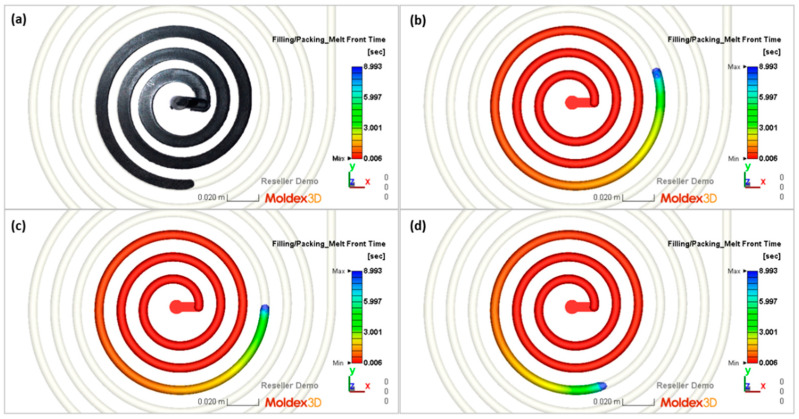
Spiral molding (part) and simulations (filling time) obtained at a screw rotation speed of N = 175 rpm and a backpressure of P_back_ = 34 MPa: (**a**) spiral molding, (**b**) A—simulation, (**c**) B—simulation, (**d**) C—simulation.

**Figure 14:**
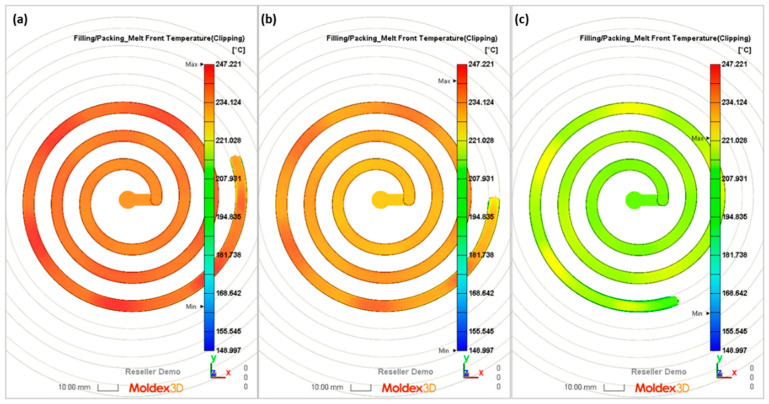
Simulations (melt front temperature) obtained at a screw rotation speed of N = 175 rpm and a backpressure of P_back_ = 34 MPa: (**a**) A—simulation, (**b**) B—simulation, (**c**) C—simulation.

**Figure 15:**
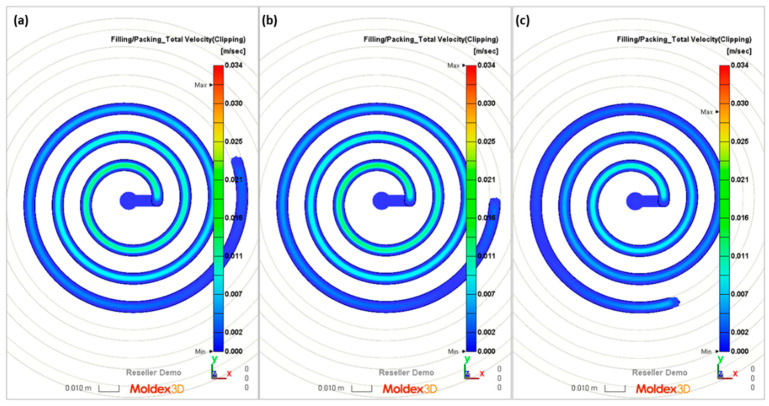
Simulations (total velocity) obtained at a screw rotation speed of N = 175 rpm and a backpressure of P_back_ = 34 MPa: (**a**) A—simulation, (**b**) B—simulation, (**c**) C—simulation.

**Figure 16:**
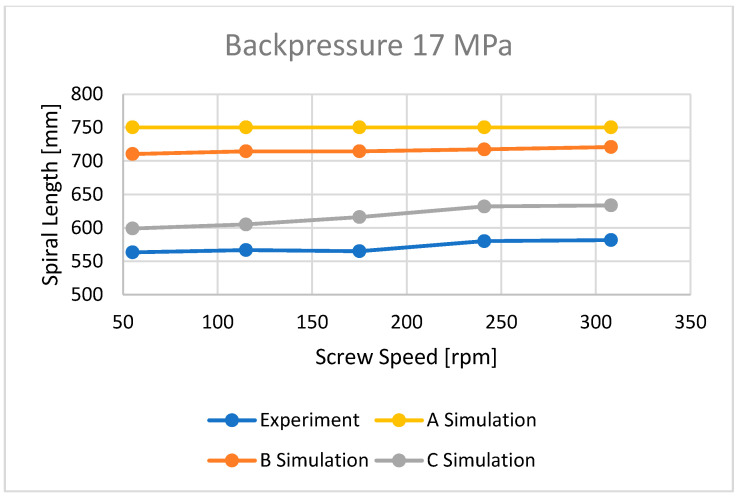
The influence of screw speed N on spiral length at a backpressure of P_back_ = 17 MPa.

**Figure 17:**
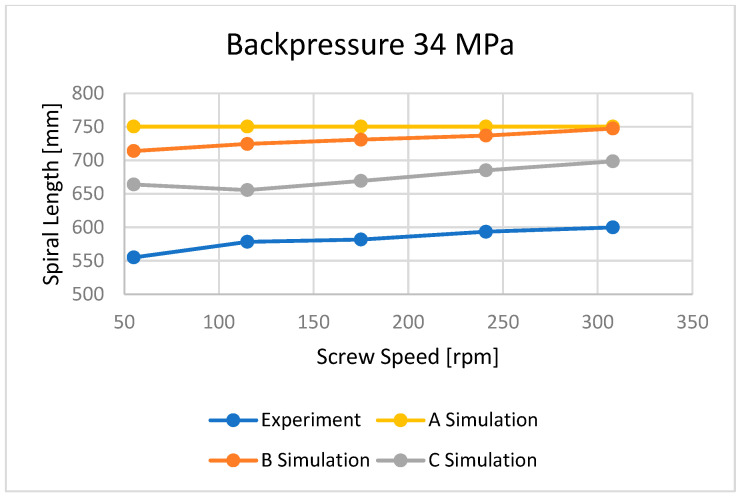
The influence of screw speed N on spiral length at a backpressure of P_back_ = 34 MPa.

**Figure 18:**
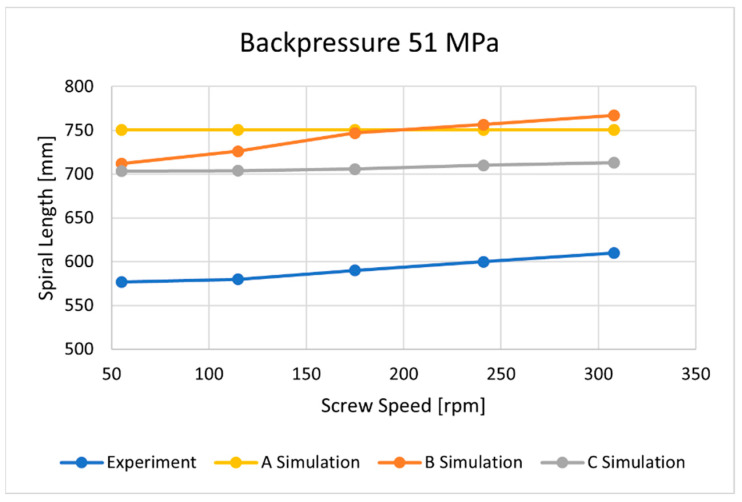
The influence of screw speed N on spiral length at a backpressure of P_back_ = 51 MPa.

The flow lengths of the material in the spiral mold, calculated using different ways to determine the temperature of the material flowing into the mold, have been compared with the results of measuring the lengths of the spirals (Figure 13, Figure 14, Figure 15, Figure 16, Figure 17 and Figure 18).

Three approaches to modeling the plastic melt flow in the mold have been tested:

A—simulations based on the recommended injection temperature given in the material card;

B—simulations based on the temperature obtained as a result of Moldex3D computations;

C—simulations based on the temperature obtained as a result of the experiment.

It is clearly seen that the best results have been obtained in the case of C—computations, i.e., based on the temperature measured. It was also observed that B—computations, i.e., based on the Moldex3D temperature simulations, give better results than A—computations, i.e., based on the data from the material card. An example of the distributions of the melt front temperature and the total melt velocity are depicted in Figure 14 and Figure 15.

## 4. Conclusions

In this research, an issue of modeling the injection-forming process has been discussed, including both the plasticizing system and mold. It has been found that a comprehensive approach to modeling this process should be considered for simulating the plastic flow in the plasticizing system and mold. The output quantities of the plasticizing system computations should be the input quantities for the mold flow computations.

Three approaches to modeling the plastic melt flow in the mold have been tested:

A—simulations based on the recommended injection temperature given in the material card;

B—simulations based on the temperature obtained as a result of Moldex3D computations;

C—simulations based on the temperature obtained as a result of the experiment.

It was found that the best results have been obtained in the case of C—calculations, i.e., based on the temperature measured. It was also observed that B—calculations, i.e., based on the Moldex3D temperature computations, give better results than A—calculations, i.e., based on the data from the material card.

It has also been concluded that the existing models of the injection-forming process have no strong experimental basis. Thus, experimentation of the material flow and melting in the injection-forming machine, which has been recently performed [45], can be a good basis for the global (comprehensive) injection-forming model. Starving, which has been observed, was not presented in the literature so far, and it may considerably influence process modeling.

Simulations performed with the use of Moldex3D software for the plasticizing system considerably improved the accuracy of modeling the flow in the injection mold. However, the modeling basics of this software are not presented in the literature [39].

The basis for modeling the plastic flow in the injection-forming machines is the solutions used in modeling the extrusion process. Recently, novel concepts of the global modeling of the extrusion process have been discussed [2]. These are based on the use of the Discrete Element Method (DEM) for modeling the solid transport and on the use of the Computational Fluid Dynamics method (CFD) for modeling the material melting and material melt flow. It seems that this novel DEM/CFD modeling concept might be applied to the global modeling of the injection process.

## Figures and Tables

**Table 1:** Material characteristics and process parameters.

Process Parameters	
Mold temperature, T_mold_	30 °C
Melt temperature, T_melt_	235 °C
Injection pressure, p_inj_	45 MPa
Filling time, t_fill_	9 s
**Material Characteristics**	
Density - Solid, ρ_s_ - Melt, ρ_m_	1120 kg/m^3^ 1020 kg/m^3^
MVR (Melt Volume Rate)	2 cm^3^/10 min (2.16 kg, 230 °C)
Heat capacity - Melt, c_pm_ - Solid, c_ps_	2320 J/(kg∙K) 1480 J/(kg∙K)
Thermal conductivity—melt, k_m_	0.240 W/(m∙K)
Melting temperature, T_m_	120 °C

## Data Availability

The data presented in this study are available upon request from the corresponding author.
